# Mycolactone: A Broad Spectrum Multitarget Antiviral Active in the Picomolar Range for COVID-19 Prevention and Cure

**DOI:** 10.3390/ijms24087151

**Published:** 2023-04-12

**Authors:** Seth Osei Asiedu, Yash Gupta, Vlad Nicolaescu, Haley Gula, Thomas R. Caulfield, Ravi Durvasula, Prakasha Kempaiah, Samuel K. Kwofie, Michael D. Wilson

**Affiliations:** 1 Department of Parasitology, Noguchi Memorial Institute for Medical Research, College of Health Sciences, University of Ghana, Legon, Accra P.O. Box GA 337, Ghana; 2 Department of Medicine, Division of Infectious Diseases, Mayo Clinic, 4500 San Pablo Road, Jacksonville, FL 32224, USA; 3 Department of Microbiology, Ricketts Laboratory, University of Chicago, Chicago, IL 60637, USA; 4 Department of Neuroscience, Division of QHS Computational Biology, Mayo Clinic, Jacksonville, FL 32224, USA; 5 Department of Biochemistry and Molecular Biology, Mayo Clinic, Rochester, MN 55905, USA; 6 Department of Biomedical Engineering, School of Engineering, University of Ghana, Legon, Accra P.O. Box 77, Ghana

**Keywords:** COVID-19, *Mycobacterium ulcerans*, mycolactone, SARS-CoV-2, viral cell entry and exit, treatment, prophylaxis, fusion protein, enveloped viruses

## Abstract

We have previously shown computationally that Mycolactone (MLN), a toxin produced by *Mycobacterium ulcerans,* strongly binds to Munc18b and other proteins, presumably blocking degranulation and exocytosis of blood platelets and mast cells. We investigated the effect of MLN on endocytosis using similar approaches, and it bound strongly to the N-terminal of the clathrin protein and a novel SARS-CoV-2 fusion protein. Experimentally, we found 100% inhibition up to 60 nM and 84% average inhibition at 30 nM in SARS-CoV-2 live viral assays. MLN was also 10× more potent than remdesivir and molnupiravir. MLN’s toxicity against human alveolar cell line A549, immortalized human fetal renal cell line HEK293, and human hepatoma cell line Huh7.1 were 17.12%, 40.30%, and 36.25%, respectively. The cytotoxicity IC_50_ breakpoint ratio versus anti-SARS-CoV-2 activity was more than 65-fold. The IC_50_ values against the alpha, delta, and Omicron variants were all below 0.020 µM, and 134.6 nM of MLN had 100% inhibition in an entry and spread assays. MLN is eclectic in its actions through its binding to Sec61, AT2R, and the novel fusion protein, making it a good drug candidate for treating and preventing COVID-19 and other similarly transmitted enveloped viruses and pathogens.

## 1. Introduction

Epidemics will always occur because of increased opportunities for greater contact between humans and pathogens of animal origins. The recent decades have witnessed several epidemic outbreaks, from Severe Acute Respiratory Syndrome (SARS), Middle East Respiratory Syndrome (MERS), HINI, and Zika virus disease to the recent COVID-19 pandemic. The COVID-19 effect was global, severely impacting the lifestyles and economies of affected nations [1], and as of 11 September 2022, 605 million confirmed cases and 6.4 million deaths have been reported globally [2]. The pandemic also witnessed the race to develop drugs and vaccines, resulting in FDA approval of some for the treatment and prevention of SARS-CoV-2 infections. However, the research and development (R&D) of these drugs and vaccines have focused mainly on the spike protein of the virus, but as observed, the virus mutates—evident from the variants that were circulating during the pandemic—which potentially may eventually render them less effective in the long term. Clinicians are overwhelmed by a complete change in viral symptoms every time there is a new strain in circulation [3,4]. Mathematical modeling has been helpful to only an extent [5,6], even with a much more extensive understanding of risk factors and prognosis [7,8]. Our group has shown the usefulness of heparin as a viral fusion inhibitor by targeting spike protein early on in the pandemic [9]. Owing to its complicated pharmacodynamics, contraindications, and lack of topical formulations [9,10,11], we have further developed lead molecules against essential SARS-CoV-2 enzymes [12] such as 3Clpro, NendoU, and Helicase [13,14,15]. An alternate approach to the COVID-19 R&D of drugs and vaccines would be blocking the host cell’s entry and exit mechanisms with safer compounds, and mycolactone (MLN) could be such a candidate [16]. In silico predictions with highly flexible and disordered viral proteins have been of limited success, and in vitro validations are of paramount importance [16,17].

MLN, a polyketide cytotoxin produced by *Mycobacterium ulcerans* [18], is responsible for painless skin ulcers, which is a major feature of the Buruli ulcer (BU) disease [19,20]. The little to no pain of the ulcers was believed to be due to the destruction of nerves in the affected areas of the skin [21,22]. Isaak et al. in 2017, among others, proposed that the lack of pain was due to its effect on sec61 [23], which suppresses inflammation, although, in another report, Song et al. reported in 2017 that this effect was rather dependent on the type-2 angiotensin II receptors (AT2R) [24]. However, in 2019 we hypothesized that the lack of pain could be due to the impairment of the human wound healing process involving the degranulation and exocytosis of the contents of red blood platelets and mast cells of infected individuals. Our reasoning was based on the observations by many BU patients who remembered previous trauma at the site of the affected areas [25,26]. Using computational methods, we showed that MLN binds strongly to the syntaxin chaperone protein Munc18b a SNARE and other proteins found in both the red blood platelets and mast cells [27]. The Munc18b protein interacts with syntaxin 11 in platelets and syntaxin 3 in mast cells [28,29]. Munc18b knockout causes a decrease in intracellular syntaxin 11 [29]; thus, the exit of granules’ contents is impaired, thereby preventing the progression of wound healing processes. We also showed that MLN binds strongly to sec61 and AT2R but discounted their effects as the primary consequence of arrested inflammatory processes that initiate wound healing processes in BU patients.

Most bacteria and viruses, including the SARS-CoV-2 virus, use the clathrin-mediated endocytosis (CME) mechanism to gain access to the interior of cells [30,31]. The process employs clathrin-coated vesicles (CCVs) formed by the assembly of clathrin-coated pits (CCPs). Both CCVs and CCPs formation require the coordination and interaction of not less than 30 proteins, each playing a critical role in one of the five stages of CCP formation: (a) initiation; (b) cargo selection; (c) clathrin coat assembly; (d) scission; (e) uncoating of clathrin [32,33]. The formation of a CCP is influenced by early acting endocytic proteins, such as FCHo 1/2, intersectins, and Eps15/Eps15R [34]. Each clathrin’s heavy chain assembles structurally to form a triskelion-like structure, with the triskelion apex composed of clathrin heavy chains (CHC). The clathrin’s three-legged structure is formed by extending these heavy chains. The clathrin N-terminal domain, which is folded into a seven-bladed-propeller, is located near the distal end of the legs, while the C terminus is located near the apex [32,35]. Clathrin-mediated endocytosis regulates the amounts of key plasma membrane proteins on the cell surface and their endocytic uptake [36]. The clathrin terminal domain (CTD) serves as the center for protein−protein interaction. CME is critical for SARS-CoV-2 infectivity [30,31], and the inhibition of CTD extremely interferes with CME [35].

Considering the eclectic nature of MLN, which includes the blockage of cellular exocytosis, we again hypothesize that MLN could also inhibit SARS-CoV-2 endocytosis, thus, the present studies. Furthermore, we also performed reverse target docking, which is used for discovering target receptors of query molecules [37], to determine if MLN has other targets within the viral proteome that could impact viral fusion and, therefore, its cell entry.

We report here the results of computational methods coupled with laboratory experiments that show MLN as a potential compound for COVID-19 treatment and prevention. We also report the discovery of a novel SARS-CoV-2 fusion protein that MLN binds to.

## 2. Results

### 2.1. MLN and Exocytosis

The detailed results relating to the binding of MLN to the studied proteins are given in Kwofie et al. 2019 [27]. Briefly, the highest binding energies (in kcal/mol) of AT2R, sec16, and Munc18b, were −9.0, −8.9, and −8.5, respectively; the rest were all below −7.0, which was considered the threshold. The Molecular mechanics Poisson-Boltzmann surface area (MM-PBSA) binding energy calculations of the MLN-Munc18b complex was done with 100 ns molecular dynamics showed an average binding energy of −247.571 kJ/mol, and that its presence elicited changes in the structural conformation of the protein. Furthermore, MLN was found to interact with Arg405, an important residue of Munc18b.

### 2.2. MLN and Endocytosis

#### 2.2.1. Molecular Docking and MLN-CTD Interactions

**Figure 1:**
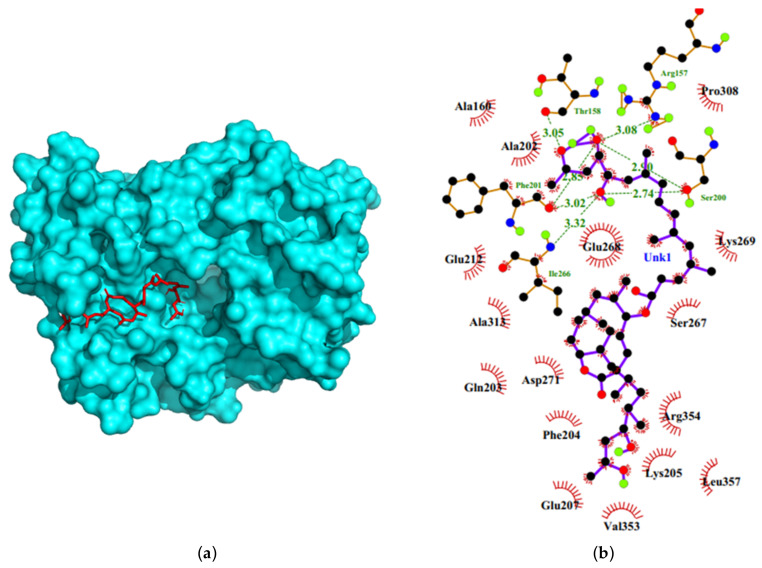
Binding mode and Ligplot+ characterization of the MLN-CTD complex. (**a**) Docking pose of MLN in one of the binding pockets of CTD. (**b**) Two-dimensional representation of the MLN-CTD protein-ligand complex. The ball and stick model of MLN compound structure has covalent bound elements depicted for different elements i.e., Carbon as black, Oxygen red, Nitrogen blue and hydrogen as green when not bound to carbon, carbon bound hydrogen not shown to avoid confusion. MLN formed seven hydrogen bonding and fourteen hydrophobic contacts.

Previous studies have used computational docking techniques to comprehend the mechanisms of novel CTD inhibitors [32]. In this current study, MLN docked firmly inside the binding pocket (Figure 1a) with a low binding energy of −9.0 kcal/mol. It also interacted with the CTD protein (Figure 1b) via hydrophobic interactions with Ala160, Ala202, Glu212, Gln203, Asp271, Glu268, Phe204, Glu207, Pro308, Lys269, Ser267, Arg354, Leu357, Val353 and hydrogen bonding with Arg157 [3.08 Å], Thr158 [3.05 Å], Phe210 [3.02 Å, 2.85 Å], Ile226 [3.32 Å], and Ser200 [2.74 Å, 2.90 Å].

#### 2.2.2. Molecular Dynamics and MM-PBSA Calculations

**Figure 2:**
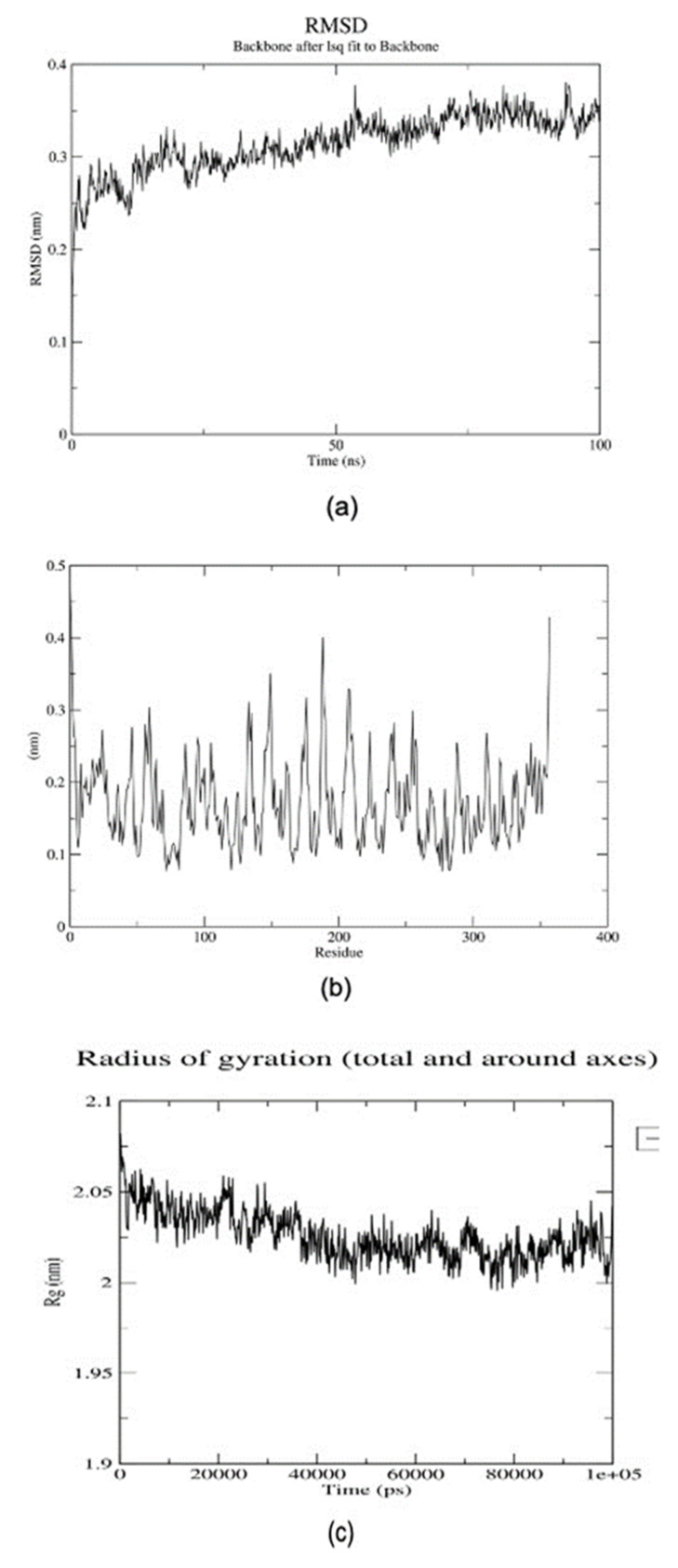
Graphical representations of Rg, RMSD, and RMSF of the MLN-CTD complex over a 100 ns MD simulation. (**a**) Graph of backbone RMSD (in nanometers (nm)) versus (time in nanoseconds (ps)). (**b**) Graph of RMSF (in nanometers (nm)) of the complex versus several residues. (**c**) The radius of the gyration graph of the complex (in nanometers (nm)) versus (time in picoseconds (ps)).

A 100 ns MD simulation was performed to understand the structural stability and conformational changes when situated under dynamic physiological conditions [38]. The parameters evaluated were the root mean square deviation (RMSD), the radius of gyration (Rg), and the root means square fluctuation (RMSF). The RMSD is a plausible measure of protein stability that accesses the deviation of the protein-ligand complex during the simulation from the initial protein backbone atomic coordinates [39]. The protein maintained an average RMSD of 0.3 nm (Figure 2a), suggesting the stability of the structure. Considering the RMSF, sizable fluctuations were observed at numerous residue positions (Figure 2b). Furthermore, the protein was stably folded per its Rg plot (Figure 2c). The Rg decreased during the first 40 ns and remained stable for the rest of the simulation time. The binding free energies of the complexes were estimated using MM-PBSA calculations. The calculations address some limitations of current scoring functions [40]. An average free binding energy of −59.210 kJ/mol was computed for the MLN-CTD complex. Energy terms, namely electrostatic, polar, non-polar, and van der Waals, contributed energies of −7.388 kJ/mol, 31.705 kJ/mol, −7.463 kJ/mol, and −76.177 kJ/mol, respectively, to the free binding energy. Additionally, a per-residue decomposition of the binding energy revealed that Met99 contributed significant energy of −5.2131 kJ/mol.

### 2.3. Reverse Target Searches for Identification of SARS-CoV-2 Proteome Binding Pockets

**Figure 3:**
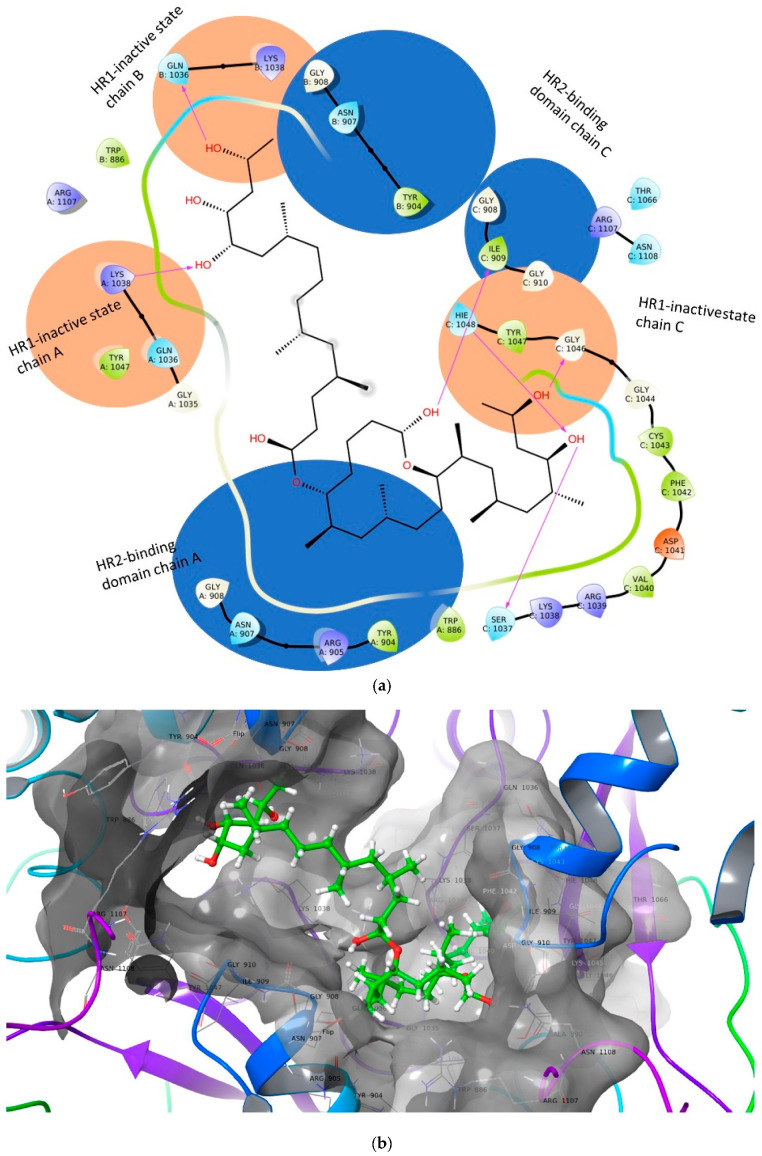
(**a**) Shows binding pocket in inactive HR1. The viral fusion domain activation involves the HR2 binding domain on HR1 to rearrange and pair with the HR2 region. (**b**) MLN A/B binding first stabilizes HR1 and second blocks the HR2 binding domain, preventing VFD activation.

Both Pharmmapper and Swiss target servers suggested the viral fusion domain (VFD) as one of the possible MLN top hits. The basis of these consensus predictions was reported with the binding of highly pharmaco-similar compounds to various VFDs. With the availability of high-resolution structures of spike proteins of SARS-CoV-2, the interaction studies were simple (Figure 3a,b). However, to stabilize crystal structures HR2 domain was truncated to avoid VFD activation and structural distortion. The heptad repeat region of the S2 domain (HR1) was intact and used to perform docking and simulation studies. Furthermore, the top-scoring complexes of wild type were subjected to 20 ns MD simulations.

**Figure 4:**
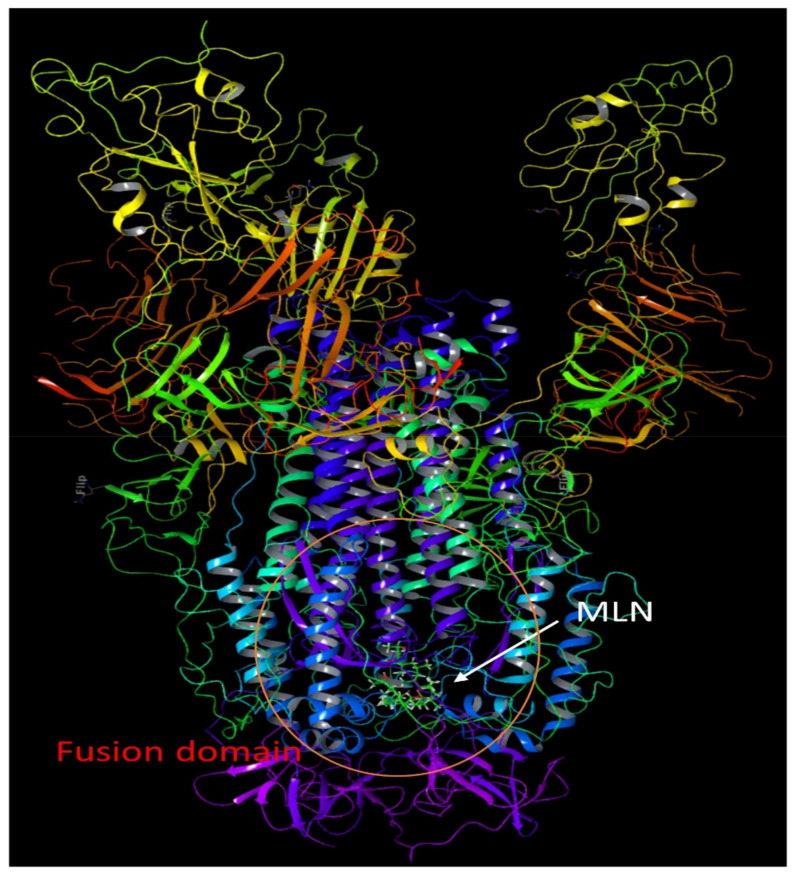
It shows a complete spike protein and MLN A/B binding pose to Heptad repeat 1 region of the S2 domain. Heptad repeats 2 region is absent in all crystalized structures as its presence destabilizes the structure.

The findings of these analyses show that the docking interactions of MLN with the three spike proteins were highly consistent owing to the highly conserved sequence and functional importance to the viral infection cycle. The MD simulation validated the high energy docking (dG_bind_ = −62.518), and the complex was highly stable throughout 20 ns MD simulations with the whole molecule involved in the interaction (Figure 4).

### 2.4. SARS-CoV-2 Inhibition by MLN and Test Compounds Using SIH Assay Immunohistochemistry

**Figure 5:**
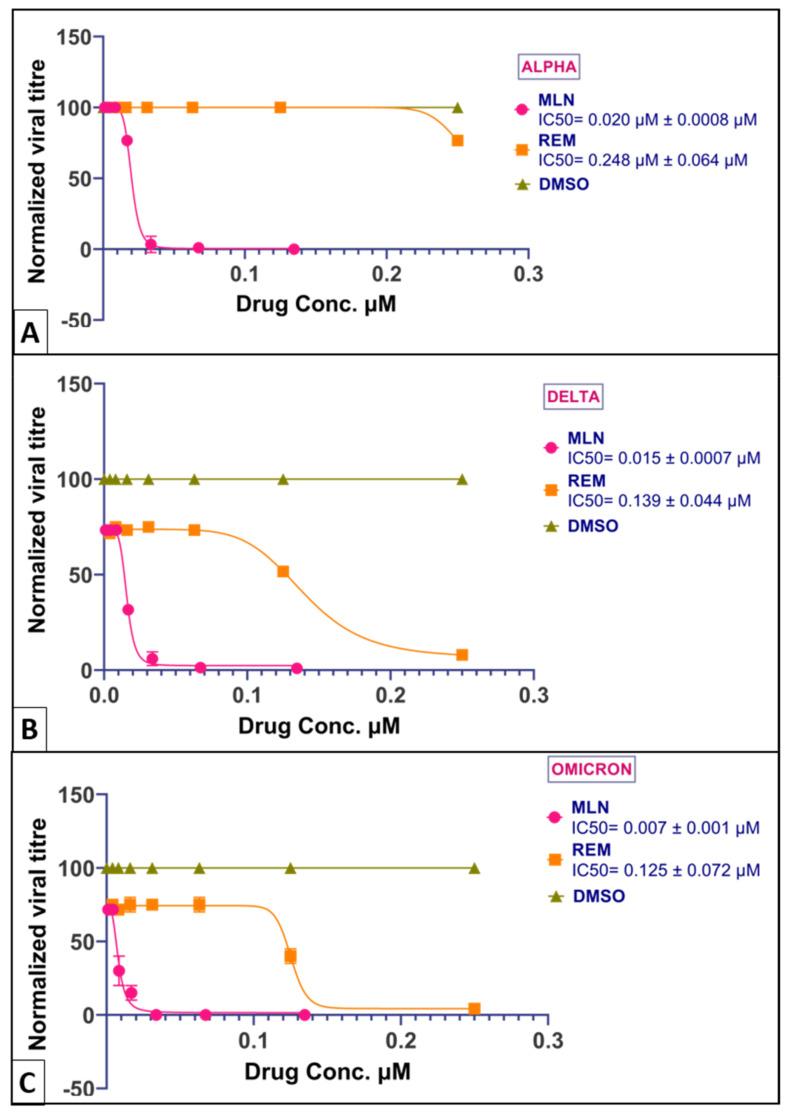
Shows the inhibition curve of MLN (hexagon data points) vs. Remdesivir (Triangular data points) against different lineages (VOCs) of SARS-CoV-2. (**A**) Alpha strain, (**B**) Delta strain, and (**C**) Omicron strain. MLN has consistent efficacy against all three VOCs.

SARS-CoV-2 inhibition by test compounds and their comparison is described in Table 1. The IC50 of MLN vs. Remdesivir (positive control) and DMSO (negative control) is described in Figure 5. 

### 2.5. Viral Entry and Spread Assays

**Figure 6:**
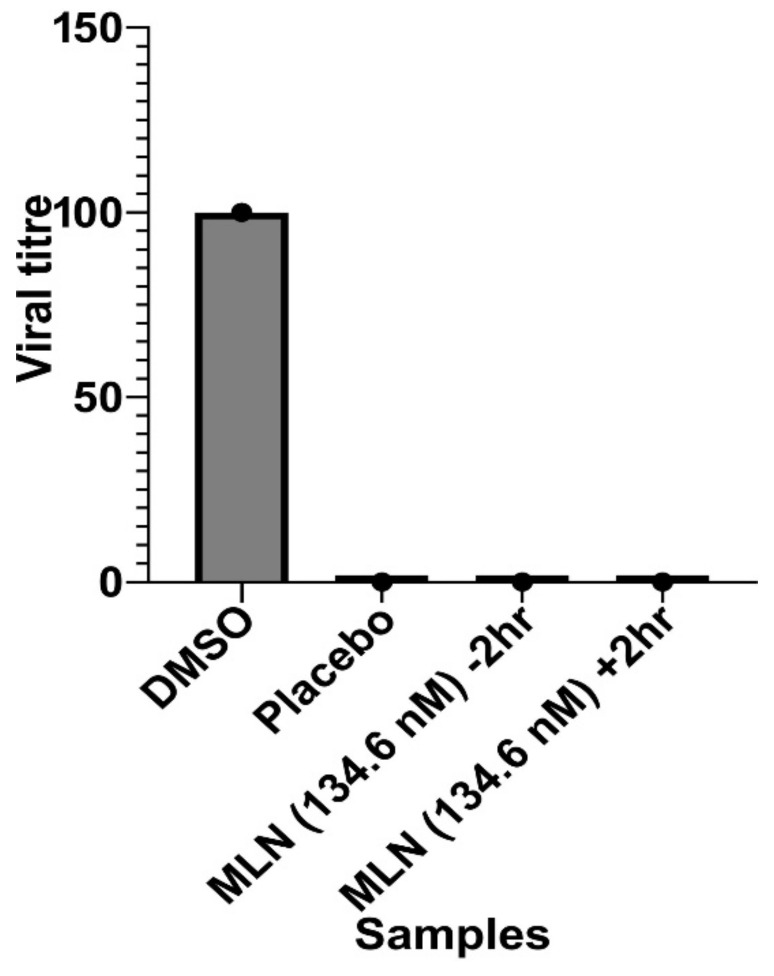
Evaluation of antiviral activities of MLN. It shows the results of entry vs. spread assays. Against virus attachment and entry/fusion, the assay was pre-treated with MLN 2 h before adding the virus to the host cell line. The experimental procedure, virus concentration (PFU/well or MOI), and the time of addition and treatment with the test compounds are presented in the method sections. Against virus replication, the same assay was conducted with a 2 h delayed drug treatment, i.e., post-introduction of viruses giving plenty of time for the viral entry. MLN has consistent activity in both assay modes. There could be multiple mechanisms complementing as this compound completely blocks the SARS-CoV-2 cycle in both assay formats.

The results are illustrated in Figure 6, which shows that MLN demonstrated consistent, complete blocking activity against the entry and spread of SARS-CoV-2.

### 2.6. MLN Cytotoxicity Evaluation

**Figure 7:**
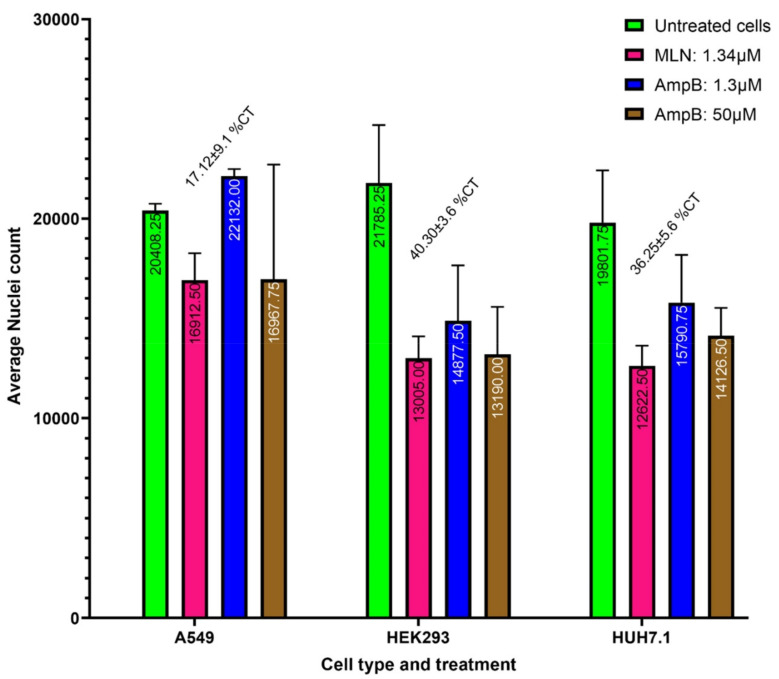
MLN cytotoxicity/cytostatic effect against different cell lines at maximum concentration tested (1.34 µM). MLN exhibited high variability in toxicity among the different cell lines. [A] Cytotoxicity (CC50 value) against Human alveolar cell line A549 was 17.12 ± 9.1%. [B] Cytotoxicity against immortalized Human fetal renal cell line HEK293 was 40.30 ± 3.6%. [C] Cytotoxicity against Human hepatoma cell line Huh7.1 was 36.25 ± 5.6%. This makes the cytotoxicity IC_50_ breakpoint ratio versus anti-SARS-CoV-2 activity more than 65×.

The cytotoxicity of MLN against Human alveolar cell line- A549 was 17.12 ± 9.1%. (Figure 7). Cytotoxicity against immortalized Human fetal renal cell line HEK293 was 40.30 ± 3.6%. [C] Cytotoxicity against Human hepatoma cell line Huh7.1 was 36.25 ± 5.6% (Figure 7). This makes cytotoxicity IC_50_ breakpoint ratio versus anti-SARS-CoV-2 activity more than 65×.

## 3. Discussion

The socioeconomic impact of the COVID-19 pandemic has engendered a race to find effective drugs in treating and arresting the spread of the virus, and our studies strongly identify MLN as just that ideal candidate compound. We used in-silico methods initially to investigate the mechanisms and followed up with laboratory experiments to validate the findings. The conventional approaches to drug discovery are very challenging, expensive, and time-consuming and need several hands. These approaches are also characterized by a low rate of therapeutic discovery, which in some cases involves bioprospecting. Moreover, the number of approved drugs in the pharmaceutical industry is steadily declining because of these challenges, with toxicity accounting for approximately one-third of withdrawals [43], and the rest are mostly due to early-stage terminations that are related to suboptimal efficacy and safety issues [44]. Repurposing existing approved drugs for COVID-19 treatment may overcome some of the huge expenditures, including remdesivir, originally designed against the Ebola virus. Another less expensive approach is using in-silico drug design methods to identify compounds in databases against viral targets, which also requires a high computing facility.

Most current approaches to anti-COVID-19 drug discovery and vaccine development, including mRNA vaccines, focus on the most obvious target, the spike protein. mRNA vaccines, for example, are made possible by designing the instructions for cells to build the unique spike protein into the vaccine. Other studies targeting the spike protein include identified anti-COVID-19 compounds in the Furin pathway [45] and associated inflammatory pathways involving MAP kinase [46]. However, the virus mutates, as evident from the variants we observe during the current pandemic. Our approach of targeting the host’s physiological pathways, which are conserved whereby mutations will be deleterious, provides us with an alternative but comes with the proviso that the identified compounds are safe.

Our previous [27] and present studies have identified MLN as a possible inhibitor of exocytosis and endocytosis, respectively. [27] showed that MLN binds strongly to proteins associated with exocytosis and the degranulation in platelets and mast cells, thereby blocking the initiation of cascading processes of wound healing to explain the painless feature of ulcers in *M. ulcerans* infections. Our present study shows that MLN is also involved in endocytosis by interacting strongly with the clathrin N terminal domain, a necessary stage of cell entry by SARS-CoV-2 [47]. We found that MLN docked firmly inside a CTD protein binding pocket with a low binding energy of −9.0 kcal/mol and that the interactions resulted from 14 hydrophobic and five hydrogen bonds (Figure 1). A 100 ns MD simulation revealed that the protein maintained an average RMSD of 0.3 nm, suggesting a stable structure with the Rg decreasing during the first 40 ns but remained stable for the rest of the 100 ns simulation time (Figure 2). Using MM-PBSA calculations, the estimated average free binding energies of the complexes was −59.210 kJ/mol for the MLN-CTD complex, and the energies contributed by the electrostatic, polar, non-polar, and Van der Waals forces were −7.388 kJ/mol, 31.705 kJ/mol, −7.463 kJ/mol, and −76.177 kJ/mol, respectively, with Met99 contributing a significant −5.2131 kJ/mol.

Our studies and others demonstrate that MLN is eclectic; thus, in addition to binding to the SNARE proteins involved in exocytosis of the platelets and mast cells via its interaction with Munc18b, endocytosis via binding to the clathrin-mediated pathway also has other effects on protein transports in cells by targeting sec61, which interferes with protein transport to the endoplasmic reticulum in eukaryotes and exocytosis in prokaryotes and the Sec61-dependent anti-inflammatory activity on the immune and nervous systems [48]. It is also a ligand that mediates K^+^-dependent hyperpolarization through AT2R activation. Furthermore, our studies have shown that it also binds to a novel virus fusion protein of the virus. Using the reverse target screening method, we also found a novel fusion protein that both MLN-A and MLN-B bind specifically to the HR1 region of the virus spike protein (Figure 3a,b). HR1 region forms a dimer with HR2 during viral envelope fusion with the target cell or endosome membrane. The binding free energies of the MLN-Spike complex at the HR1 region were very high, with an average global dG bind of −62.518, and the ligand-protein complex was also highly stable throughout 20 ns MD simulations (Figure 4). The cumulative multitarget activity not only makes this agent highly effective it also dramatically decreases the probability of resistance.

The symptoms associated with severe COVID-19 disease include hyperinflammation, blood clots that surround internal organs [49], and thrombocytopenia or low platelet counts [50]. The high-risk group of severe COVID-19 disease includes cases with underlying conditions of diabetes and hypertension, and they are also at a comparatively high risk of mortality [51,52]. All of these conditions suggest the involvement of red blood platelets in the etiology of severe COVID-19. The activation of platelets results in the degranulation of both α (alpha) and dense granules, which contain factors that initiate wound healing processes. Principal among these factors is the adhesive glycoproteins, coagulation factors, von Willebrand factor, mitogenic factors, and vascular endothelial growth factors in the α granules. The contents of the dense granules include serotonin, calcium, ATP/ADP, vascular permeability factor, and chemokines [53]. All these factors from the platelets and others from the mast cells are released into the microvasculature to initiate the cascade of wound healing processes of four overlapping phases; hemostasis which is the process of the wound being closed by (1) clotting, (2) inflammatory, (3) proliferative, and (4) maturation. Thus, platelet involvement appears as a characteristic corollary of the severe symptoms associated with COVID-19 [54]. Studies have also shown that platelets facilitate the uptake of SARS-CoV-2 secretion of the subtilisin-like proprotein convertase furin [54]. Since platelet factors are released into the bloodstream through exocytosis, MLN could also be considered for use to manage the severe form of the disease.

Our preliminary experiments showed that MLN achieves 90% inhibition at 20 nM and 100% inhibition at 42 nM. When we compared the inhibition of MLN with those of currently FDA-approved drugs Molnupiravir and PF-00835231, the 80% inhibition achieved was 0.03 µM, 0.30 µM, and 0.68 µM, respectively (Figure 5 and Figure 6). We went further to test the efficacies (viral titer reduction) of MLN and remdesivir against the alpha, delta, and Omicron strains of the virus and found MLN was still comparably efficacious at 0.02 µM, 0.015 µM, and 0.007 µM compared to 0.248 µM, 0.139 µM, and 0.125 µM for remdesivir (Figure 5). Furthermore, MLN also exhibited complete prevention of entry and spread of the virus. It is worth noting that although MLN also binds to sec16, so far, none of the numerous sec16 inhibitors that have been reported is active in the therapeutic range of >20 times their toxicities.

With regards to safety, our in vitro studies against human cell lines demonstrate that MLN is not cytotoxic (Figure 7), and Babonneau et al. (2019) have demonstrated that upon intradermal injection of MLN, the half-life of MLN in the periphery blood circulation is short and that the little amounts that remain in tissues provide a long-lasting effect [55].

Thus, MLN could potentially be a preventive drug that blocks the entry, in vivo replication, and the spread of SARS-CoV-2 and diminishes inflammation, thus impacting severe COVID-19 morbidity. We see here the expanded potential of MLN effects on other viral diseases of public health importance, dengue, ZIKA, yellow fever, etc., and other non-viral pathogens that require the host’s cell entry and exit, replication, and hyper-inflammation.

## 4. Materials and Methods

### 4.1. MLN and Exocytosis

The detailed computational methods employed to investigate the inhibition of exocytosis in platelets and mast cells are fully described by [12]. Briefly, molecular dockings of MLN to proteins were performed, and molecular mechanics Poisson-Boltzmann surface area (MM-PBSA) binding energy calculations of MLN and Munc18b complex were done with 100 ns molecular dynamics simulations. The target proteins were the soluble n-ethylmaleimide-sensitive factor attachment protein receptor (SNARE), the vesicle-associated membrane protein 8 (VAMP8), synaptosomal-associated protein (SNAP23), syntaxin 11, Munc13-4 in mast cells (its isoform Munc13-1 was used), and Munc18b, and other published known MLN targets sec61, angiotensin II type 2 receptor (AT2R), and Wiskott–Aldrich Syndrome protein (WASP) [23].

### 4.2. MLN and Endocytosis

#### 4.2.1. Protein Structure Retrieval and Preprocessing

The experimentally solved three-dimensional (3D) structure of the clathrin-terminal domain (CTD) was retrieved from the Protein Data Bank [24] with accession number PDB ID: 2XZG. The removal of available water molecules and ligands was done in PyMOL v 4.0.0 [25]. The structure was then minimized in GROMACS v 2018 [26] using the steepest descent algorithm at 50,000 steps. GROMOS96 43a1 force field was used to generate the protein topology and position restrain files. Periodic Boundary Conditions (PBC) were applied to the structure with the protein centered 1 nm from the edge of a cubic box to monitor the movement of all particles and avoid edge effects on the surface atoms [27]. The resulting structure was solvated with SPC water [27] and neutralized with Na and Cl atoms.

#### 4.2.2. Molecular Docking of MLN to CTD

AutoDock Vina [28] in PyRx [29] was used to dock MLN against the minimized CTD protein structure. The .sdf format of MLN with ID: 5282079 was retrieved from PubChem [30] and imported into OpenBabel [31]. It was then minimized using the Universal Force Field (UFF) for 200 steps and optimized using the conjugate gradient. A grid box of dimensions 63.589, 59.346, 53.464 Å and center 44.251, 44.1745, 44.3269 Å that covered the entire protein surface was set for docking. Additionally, a default exhaustiveness of eight was used.

#### 4.2.3. Molecular Dynamics Simulations of MLN-CTD Complexes

One hundred ns MD simulations of the MLN-CTD complex were performed using GROMACS v 2018. The protein topologies were initially generated using the GROMOS96 43a1 force field [32] and the ligand topologies via the PRODRG server [33]. A complex was formed by merging the topologies. The complex was then solvated with water molecules in a cubic box of size 1.0 nm^3^ and neutralized with Na and Cl ions. Energy minimization of the complex was conducted for 50,000 steps using the steepest descent algorithm. Mycolactone was restrained before the constant-temperature, constant-volume (NVT), and constant-temperature, constant-pressure ensemble (NPT) simulation. Equilibration of each complex was performed for 100 ps apiece, and the final MD simulation was conducted for 100 ns with time steps of 2 fs under particle mesh Ewald (PME). The free binding energies were calculated using g_mmpbsa [34]. The binding free energy contribution per residue was calculated using MM-PBSA, and the output plots were generated with R.

### 4.3. Reverse Target Searches for Novel Binding Pocket

The 3D model structures of both MLN A and B were predicted using the steepest descent algorithm with force field UFF [35], where all atoms move in Avogadro software v. 1.2. MLN predicted target pool was analyzed by a battery of servers in search of possible targets using TargetHunter, Pharmmapper, Spider, SuperPred, Stitch, Hitpick, reversescreen3D, and Swiss target prediction to compare this with the mutant viral spike proteins, Wild type (PDB id: 7TAT), Delta-Plus (PDB ID: 7W9E), and Omicron (PDB id: 7Q07) was docked with both MLN A and B using the glide module of Schrodinger software v. 2022-3 and XP scores were calculated. Further, the top-scoring complexes of wild type were subjected to 20 ns MD simulations.

### 4.4. Experimental Studies

#### 4.4.1. SARS-CoV-2 Strains and Cell Lines

Human alveolar basal epithelial A549-ACE2 [36] cells and SARS-CoV-2 [novel coronavirus (nCoV)/Washington/1/2020] provided by N. Thornburg (CDC) via the World Reference Center for Emerging Viruses and Arboviruses (Galveston, TX, USA) and from BEI Resources. Variants of concern were obtained from BEI resources. Delta Variant (BEI Cat.ID. NR-55671) Isolate hCoV-19/USA/MD-HP05285/2021 (Lineage B.1.617.2) contributed by Andrew S. Pekosz. Omicron Variant (BEI Cat.ID. NR-56461) Isolate hCoV-19/USA/MD-HP20874/2021 (Lineage B.1.1.529) also contributed by Andrew S. Pekosz.

#### 4.4.2. Antiviral Inhibition Assays

##### Evaluation of Viral Inhibition by MLN Using Spike Immunohistochemistry Assay

All SARS-CoV-2 infections were performed under biosafety level 3 conditions on the human cells in DMEM supplemented with 2% fetal bovine serum (FBS) and antibiotics. For the preliminary selection of hits, cells were pre-treated with MLN or other inhibitors for 2 h with 2-fold dilutions beginning at 50 μM in triplicate for each assay. To enumerate the IC_50_ or percent inhibition, an identical treatment was performed with 10-fold dilutions beginning at 50 μM (134.6 nM in the case of MLN). A549-ACE2 cells were infected with a multiplicity of infection (MOI) of 0.5 in media containing the appropriate concentration of drugs. After 48 h, the cells were fixed with 10% formalin, blocked, and probed with mouse anti-Spike antibody (GTX632604, GeneTex, Irvine, CA, USA) diluted 1:1000 for 4 h, rinsed, and probed with an anti-mouse- horseradish peroxidase (HRP) for 1 h, washed, then developed with 3, 3′ Diaminobenzidine (DAB) substrate for 10 min. Spike-positive cells *(n* > 40) were quantified by light microscopy as blinded samples.

##### Evaluation of Viral Inhibition by MLN Using Plaque Assay

Viral titers were determined by plaque assay. Briefly, a monolayer of cells was infected with serial dilutions of virus samples for 1 h at 37 °C. The viral inoculum is then removed and replaced by a MEM overlay media containing 1.25% carboxymethyl cellulose. Cells were incubated for 72 h, after which the overlay media was removed, and cells were fixed with 10% formalin and stained with 0.25% crystal violet solution. Plaques are then counted, and the viral concentration is calculated using the following method. The average value of plaques in replicate wells × dilution factor ÷ virus inoculum volume (in mL) = titer in PFU/mL. The data were analyzed and plotted using GraphPad Prism v. 9.5.1. (GraphPad Software Inc., San Diego, CA, USA), and IC_50_ values were extrapolated from the nonlinear fit of the response curves.

##### MLN and Virus Entry and Spread Inhibition Assay

A549-ACE2 cells were treated with 134.6 nM MLN (~100-fold higher than IC_50_) 2 h before infection with SARS-CoV-2 and 2 h after infection with SARS-CoV-2 with a protocol modified from Chianese et al., 2022 [56]. This experiment assessed whether MLN would block the entry of the virus to cells and, if the virus gets infected, whether it blocks the re-entry/spread to neighboring cells. Cells were infected with an MOI of 0.5 for 2 h. Then, the infection medium was replaced with a medium containing MLN or dimethyl sulfoxide (DMSO as vehicle control), and the samples were incubated at 37 °C for 24 h. The plates were probed by mouse anti-Spike antibody (GTX632604, GeneTex, Irvine, CA, USA) and read as described earlier.

#### 4.4.3. Cytotoxicity Assays

To confirm if the MLN has no adverse effect on the host cells, we conducted cytotoxicity tests on various cell lines, primarily the lung epithelium, kidney, and liver, i.e., A549, HEK293, and HUH7.1 cell lines. MLN cytotoxicity/cytostatic effect against different cell lines could only be tested at a maximum concentration of 1.34 µM again due to a dilute stock source in 96-well format. A549, HEK293, and HUH7 cells were maintained in filter cap cell culture flasks at 37 °C in a humidified atmosphere containing 5% CO_2_ and Dulbecco’s modified Eagle’s media supplemented with 10% fetal bovine serum and 1% penicillin-streptomycin (Gibco Life Technologies, Cergy-Pontoise, France). A549, HEK293, and HUH7 cells were seeded in separate plates in a 96-well black clear bottom cell culture grade plate at a density of 5000 trypsinized cells/100 L/well; cell counting was performed by trypan blue (sigma) live cell staining and automated counting by Invitrogen Countess 3 automated cell counter. After adding various test drugs/compounds to the test plates, Amphotericin B as a positive control and DMSO vehicle (same as sample volume 1 L as the negative control), the plates were incubated for 24 h at 37 °C and humidified at 5% CO_2_. Compounds were dissolved in cell culture-grade DMSO (stock concentration: 10 mM; highest concentration: 100 mM; 100× diluted for other compounds and 1.34 µM for MLN). The highest concentrations were serially diluted by a factor of 2:1 10 times, with each series being carried out in triplicates. The Hoechst 33,342 dye from Thermo Fisher, Waltham, MA USA was used to stain the incubated plates, which were then incubated for 24 h at 37 °C with 5% CO_2_ and humid conditions. The cells were then imaged using a 4× plan fluor objective (4× Plan Apo Lambda Nikon air objective lens with a camera binning of 2 and a pixel size of 3.367 m × 3.367 m) with bright field and LED illumination capture DAPI channels by Sony CMOS inbuilt camera on the ImageXpress Pico High-Content Imaging System Microscope from Molecular Devices, San Jose, CA, USA. Additionally, automated data processing tools with pre-configured analysis algorithms were used to process the imaging data, and nucleus counts were recorded. GraphPad prism software version 9.5.1 was used to perform Nonlinear regression (curve) utilizing normalized values on the Y-axis and log transformed drug concentrations on the X-axis to quantify cell death and determine CC50 of test compounds. The maximum drug concentration effect on cells was used to make the graph for maximum toxicity observed, and corresponding ratios with average IC_50_s against different strains were calculated.

## 5. Patents

Part of this work is protected under Provisional Patent Doc# 1152-001 PROV. “Use of mycolactone (MLN) and Derivatives Thereof for Treatment of Microbial Infections.”

## Figures and Tables

**Table 1:** Comparative potency breakpoints of leading antiSARS-CoV-2 agents in comparison with MLN.

Name	IC-50, Anti-SARS-CoV-2 (µM)	80% Inhibition of SARS-CoV-2 (µM)	Reference/Justification
ß-D-N4-hydroxycytidine (molnupiravir)	0.10 ± 0.06	0.30 ± 0.09	[41]
PF-00835231 (Pfizer)	0.23 ± 0.03 (with P-gp efflux inhibitor)	0.68 ± 0.023	[42]
MLN	0.02 ± 0.006	0.03 ± 0.005	0.269 mM stock, Figure 5 and Figure 6

## Data Availability

Not applicable.
